# Determination of cystatin C reference interval for children in Croatia

**DOI:** 10.11613/BM.2024.010702

**Published:** 2023-12-15

**Authors:** Vlasta Cigula Kurajica, Željka Vogrinc, Ana Turčić, Slobodan Galić

**Affiliations:** 1 Department for Laboratory Diagnostics, University Hospital Centre Zagreb, Zagreb, Croatia; 2 Department of Pediatrics, University Hospital Centre Zagreb, Zagreb, Croatia

**Keywords:** cystatin C, children, immunoturbidimetry, reference value

## Abstract

**Introduction:**

Cystatin C is considered an early marker of kidney damage. The aim was to determine the reference interval in children since this information was not available from the test manufacturer.

**Materials and methods:**

Included were children aged 0 to 18 years undergoing routine check without history of any renal disease. Cystatin C was measured by the immunoturbidimetric method, and creatinine by the enzymatic method on a Cobas c501 analyzer (Roche Diagnostics, Manheim, Germany). Reference intervals were determined according to the CLSI C28-A3 guidelines using a robust method and a nonparametric percentile method, depending on the sample size. The Schwartz’s formula was applied to estimate glomerular filtration (eGFR) from cystatin C.

**Results:**

The cystatin C reference interval for children aged 1-18 years (N = 204, median 8 years) was from 0.61 mg/L (90% CI: 0.53 to 0.64) to 1.08 mg/L (90% CI: 1.07 to 1.14). Differences according to sex were not found. For children aged 0-1 years (N = 29, median 5 months), the reference interval was from 0.60 mg/L (90% CI: 0.48 to 0.72) to 1.49 mg/L (90% CI: 1.36 to 1.61). The sample size was too small to test the difference according to sex. The eGFR was 76 (70-88) mL/min/1.73m^2^ for males and 83 (74-92) mL/min/1.73m^2^ for females.

**Conclusion:**

The cystatin C reference intervals for Croatian pediatric population according to age were determined. The cystatin C concentrations in children reach adulthood values after the first year. The cystatin C Schwartz’s formula is applicable for eGFR calculation in children.

## Graphical abstract

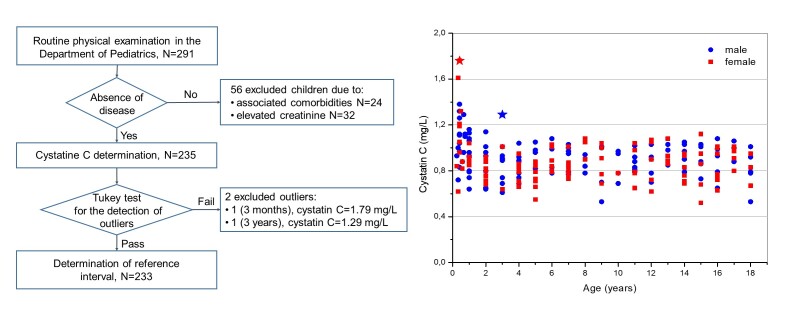

## Highlights

The cystatin C reference interval for children aged 1-18 years was 0.61-1.08 mg/L

The eGFR was 70-88 mL/min/1.73m^2^ for males and 74-92 mL/min/1.73m^2^ for females

## Introduction

The most reliable test for assessing the overall kidney capacity for early detection of acute kidney diseases and for determining the stage of chronic kidney disease in all age groups is the glomerular filtration rate (GFR) (*1*). In everyday clinical practice, the assessment of GFR is most often accomplished by determination of serum creatinine concentration or determination of endogenous creatinine clearance. However, serum creatinine is not an ideal biomarker of impaired kidney function as its formation is influenced by numerous factors like diet, muscle mass, muscle metabolism, sex following puberty *etc.* (*1*). Additionally, creatinine has a small intraindividual biological variability (CVi 4.5%) and a large interindividual biological variability (CVg 14.1%) resulting in extremely small individuality index (II = 0.32). This means that the clinically significant changes in plasma creatinine concentration can occur even when creatinine is within reference intervals, especially during acute conditions (*2*, *3*). Furthermore, creatinine clearance usually requires longer periods of urine collection, from 12 to 24 hours and also could be inaccurate, either because of inadequate urine collection or because of the tubular secretion of creatinine in the kidney (*4*). For all the above stated reasons, creatinine is not an appropriate marker of renal function in children. Furthermore, in the first days of life (up to 72 hours after birth) serum creatinine is elevated due to the balance of creatinine in the placenta, and it reflects the function of mother’s kidneys. In other words, the value of serum creatinine in newborns corresponds to the value of serum creatinine of the mother (*2*). Because creatinine reference values for adults are higher than reference values for neonates, the determined values for neonates are apparently elevated, making estimation of renal function in neonates based on serum creatinine unsatisfactory. Creatinine concentration decreases during the following weeks of a newborn’s life and gradually better reflects the child’s kidney function (*2*, *5*).

Serum cystatin C has been shown to be a better and more reliable marker of renal function and several studies have shown that serum cystatin C correlates with glomerular filtration equal to or better than creatinine (*6*, *7*). Cystatin C is a protein of small molecular mass (13.3 kDa) consisting of 120 amino acids. It is an inhibitor of cysteine protease and is synthesized in all cells with a nucleus. Its synthesis is constant and it is found in various body fluids, including blood plasma. As a small basic protein, cystatin C is eliminated from the circulation exclusively by glomerular filtration, is reabsorbed and degraded in the proximal tubular cells of the kidney, without significant tubular secretion, and is therefore a good indicator of glomerular filtration (*8*). Unlike creatinine, the concentration of cystatin C does not depend on sex, age, weight or constitution and is not affected by loss of muscle mass, anorexia, neuromuscular disease and vegetarian diet (*9*). However, it should be noted that high doses of glucocorticoids increase the formation of cystatin C, as well as that cystatin C values are reduced in hypothyroidism and elevated in hyperthyroid states (*10*, *11*). Based on cystatin C concentration, acute renal failure can be diagnosed earlier than by determination of serum creatinine, and serum cystatin C is certainly a more sensitive marker of changes in GFR than serum creatinine (*12*, *13*).

The cystatin C molecule does not cross the placenta, so the determined value is the actual value of the newborn’s cystatin C and thus a better biomarker of renal function in newborns (*2*, *8*). Furthermore, while creatinine concentrations in the serum of children increase until adulthood, the concentration of cystatin C is constant after the first year. The constant values after the first year of life until adulthood make cystatin C a more reliable marker for identifying abnormal kidney function in children compared to creatinine.

The assessment and monitoring of renal function through GFR is based on creatinine concentration. For children aged 1 to 18 years, Croatian national recommendations require the mandatory use of the enzymatic method for measuring serum creatinine concentration, since this method is traceable to isotope dilution mass spectrometry (IDMS). Also, to estimate GFR from creatinine concentration, Croatian national recommendations suggest the use of the Schwartz equation (*14*). This equation for estimating GFR is considered unreliable for small children, and has limitations in cases of overweight, malnutrition, physical deformities, as well as in the case of acute kidney failure (*15*). However, due to its simplicity, the Schwartz equation is still the most commonly used predictive formula for estimating renal function in children based on serum creatinine (*16*). Another approach involves use of exogenous tracer such as iohexol but its disadvantage is the need for multiple blood draws (*17*).

Given the numerous limitations of GFR determination methods, the use of cystatin C to estimate GFR is increasing. Accordingly, new equations for estimating GFR have been developed that are based on the determination of cystatin C. Among these equations, there are significant differences in specificity and accuracy. For the general pediatric population the most appropriate is considered the equation also defined by Schwartz *et al*. (*18*). This equation is considered to more accurately estimate GFR for high-risk kidney disease groups such as patients with reduced muscle mass, oncology patients and hematopoietic stem cell transplant recipients, than equations based on creatinine (*19*-*21*).

Since there are no reference intervals for cystatin C for the population of children in Croatia, the aim of this study was to determine the reference values for cystatin C for children aged 0 to 18 years according to the CLSI C28-A3 guidelines (*22*). Additionally, we wanted to determine reference values for estimated glomerular filtration rate (eGFR) based on cystatin C values using the Schwartz equation.

## Materials and methods

### Subjects

**Figure 1:**
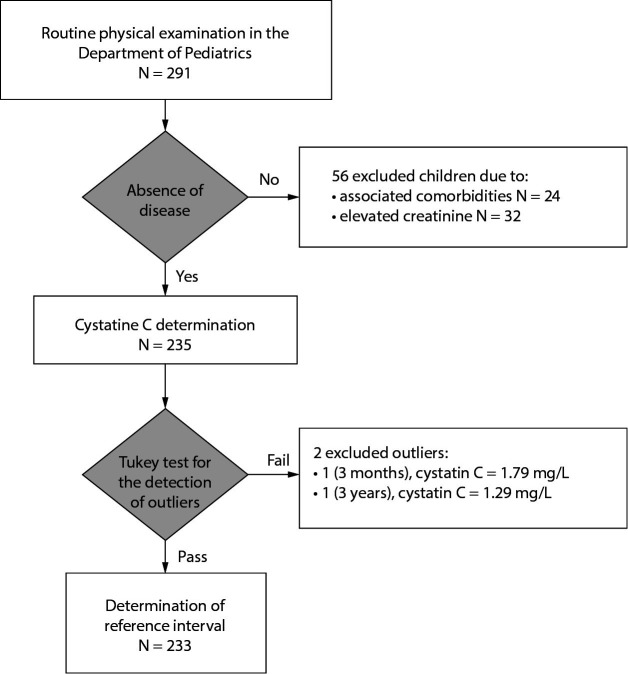
Flowchart of subject recruitment and exclusions.

Included were children aged 0 to 18 years in a stable condition, without being on any therapy and undergoing routine check at the Department of Pediatrics, University Hospital Centre Zagreb. The exclusion criteria were: the history of renal disease or the presence of comorbidities connected with renal disease, an acute illness within the past month and elevated creatinine, as shown in the Figure 1. Children were prospectively included between October 2017 to January 2018 and the residual serum samples were used for cystatin C measurement. Venous blood sampling has been accomplished according to Croatian national recommendations (*23*). The study was approved by the Institutional Ethics Committee, No 02/21 AG.

### Methods

Serum samples were taken in tubes with inactive resin and a clotting activator (Greiner Bio One Vacuette, St. Gallen, Switzerland). Cystatin C was measured by the immunoturbidimetric method, using the reagent Tina-quant cystatin C (generation 2) (Roche Diagnostics, Manheim, Germany). The method is standardized according to the certified international standard for calibrators ERM-DA471/IFCC (*24*). The primary measurement range for cystatin C determination with this reagent is 0.40-6.80 mg/L. Analytical performance of the cystatin C assay was evaluated using the internal quality controls (IQC) cystatin C Control Set with three control levels (Roche Diagnostics, Manheim, Germany).

Creatinine concentration was determined by an enzymatic photometric method with creatininase, traceable to IDMS and National institute of standards and technology (NIST) secondary reference material SRM 967, with an age-appropriate reference range. The analytical performance was evaluated using two IQC PreciControl ClinChem Multi 1 and 2 (Roche Diagnostics, Manheim, Germany).

Both analytes were determined on a biochemical analyzer Cobas 6000 c501 (Roche Diagnostics, Mannheim, Germany) and were twice a year included in the external quality assessment organized by INSTAND, while creatinine is also included in the national quality control program, CROQALM.

The coefficients of variation of internal quality control for cystatin C ranged from 2.10% to 3.70%, and for creatinine assay were 1.60% and 1.78%. For both assays, all submitted results for external quality assessment met the criteria of the external organizer.

Reference intervals for cystatin C were determined according to CLSI C28-A3 guidelines (*22*). All values were visualized on an age to cystatin C concentration plot and marked according to sex to visually distinguish whether there is a need to establish separate reference cystatin C intervals based on age. The selected ranges were tested for differences in age and sex by Harris and Boyd test. If a calculated z-score was above the critical (z*) value, the separate reference intervals were calculated for tested subgroups (*22*, *25*). The Tukey test was performed for the detection of suspected outliers which were excluded for reference cystatin C interval determination. All outliers were excluded to maximize the biomarker sensitivity as the use of gold standard test for excluding impaired kidney function was not justified in the pediatric population (Figure 1). A nonparametric percentile method was used for the calculation of cystatin C reference intervals in a group with more than 120 subjects, while in the group of less than 120 subjects a robust method according to Horn and Pesce was used (*26*). Confidence interval was set to 90% since it is considered as acceptable and recommended reference interval in biomedical literature.

The glomerular filtration rate was calculated using Schwartz equation based on cystatin C: eGFR = 70.69 (CysC)^-0.931^, where eGFR is the estimated value of GFR in mL/min/1.73 m^2^, and CysC is the serum cystatin C concentration in mg/L (*18*).

### Statistical analysis

Quantitative data were tested for normality using the Shapiro-Wilk test and presented with mean and standard deviation (SD) if normal, and median and interquatile range if they followed non-normal distribution. Age was presented with median and minimum and maximum range values. Categorical data were presented with percentages or ratios in case of below 30 subjects. The differences in eGFR between groups were tested with Mann-Whitney test. The P < 0.05 was considered statistically significant.

Graph visualization and the calculations according to Harris and Boyd were performed in Microsoft Office Excel 2000 (Microsoft Office, Redmond, USA), while the rest of statistical analysis was performed in MedCalc, version 20.112 (Medcalc Software, Mariakerke, Belgium) statistical software.

## Results

**Figure 2:**
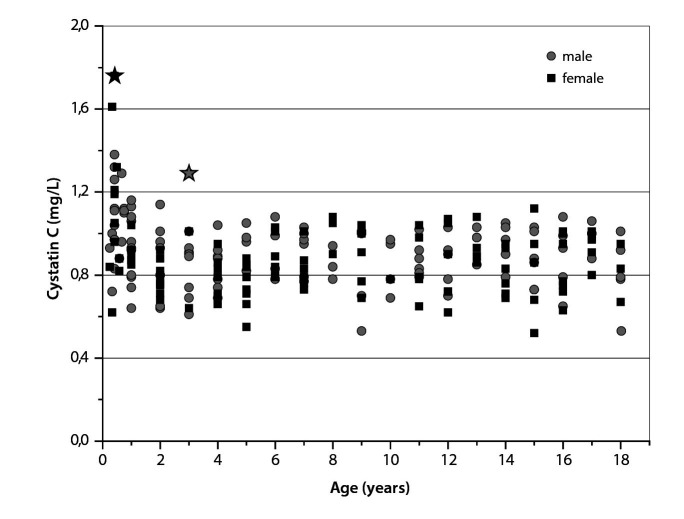
Cystatin C values according to age and sex. Males are presented with black squares and females with grey circles. Stars indicate outliers excluded from reference intervals calculation.

Altogether 233 children were included for reference intervals determination, age 6 years (3 months to 18 years), of which 53% were boys. Cystatin C values according to age and sex are presented in Figure 2. The determined pediatric cystatin C reference intervals according to age are shown in Table 1. In the younger than 12-month group 29 children (18 boys) were included, age 5 months (3 to 9 months). A cystatin C reference interval 0.60 mg/L (90% CI: 0.48 to 0.72) to 1.49 mg/L (90% CI: 1.36 to 1.61) was obtained, with a median of 1.05 (0.92-1.19) mg/L.

**Table 1:** Cystatin C reference intervals

**Age** **median (min - max)**	**N (m)**	**Method**	**Lower limit** **(90% CI) mg/L**	**Upper limit** **(90% CI) mg/L**
5 months (3-9)	29 (62)	Robust method (CLSI C28-A3)	0.60 (0.48 to 0.72)	1.49 (1.36 to 1.61)
8 years (1-18)	204 (52)	Non-parametric percentile method (CLSI C28-A3)	0.61 (0.53 to 0.64)	1.08 (1.07 to 1.14)
The examined population consisted of 233 children with the absence of kidney disease based on the doctor’s medical examination and serum creatinine values within reference intervals. CI - confidence interval. m - the percentage of males in the group.				

In the participants older than 1 year, 204 children (52% boys) were included for cystatin C reference intervals calculation, age 8 years (1 to 18 years). The established cystatin C reference interval was 0.61 mg/L (90% CI: 0.53 to 0.64) to 1.08 mg/L (90% CI: 1.07 to 1.14), median 0.86 (0.77-0.97) mg/L.

**Figure 3:**
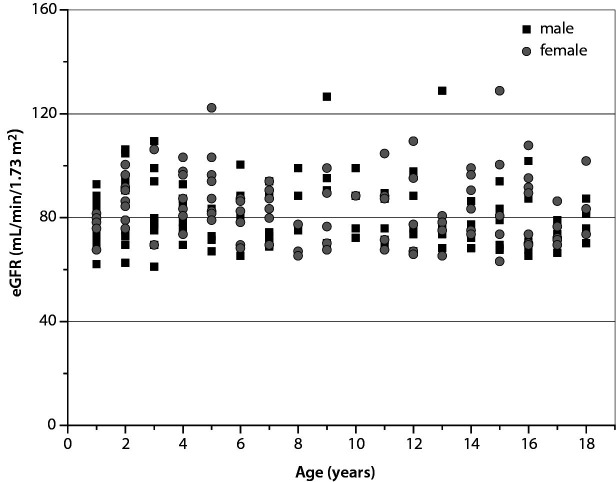
Estimated glomerular filtration rate (eGFR) based on Schwartz equation according to age and sex. Males are presented with black squares and females with grey circles.

Schwartz cystatin C equation (Figure 3) revealed differences in eGFR for males 76 (70-88) mL/min/1.73m^2^ and females 83 (74-92) mL/min/1.73m^2^ (P = 0.004).

## Discussion

The obtained reference values of cystatin C for children up to 1 year and from 1 to 18 years, are in accordance with the research of other authors who used other, non-standardized or non-certified calibrators in commercial tests (*9*, *27*, *28*).

The obtained reference values of cystatin C for children under the age of 1 are higher than the values for children aged 1 to 18 years. According to Boenkamp *et al.* the reference interval of cystatin C for children up to the fourth month of life is 1.64-2.59 mg/L, and for children older than one year it is 0.70-1.38 mg/L (*9*). According to Fischbach *et al*. the mean value of cystatin C in serum using the immunonephelometric method for infants up to 18 months of age is 0.70-1.18 mg/L, while for children older than 18 months it is 0.44-0.94 mg/L (*27*). Higher values of the cystatin C reference interval in serum for children in the first year of life, especially in the first month, reflect a low rate of glomerular filtration due to kidney immaturity (*9*, *27*). As the kidneys mature, the concentration of serum cystatin C stabilizes and remains constant until the age of 18 (*6*, *9*, *28*, *29*). In the present study, available were a relatively small number of samples of children under the age of one year and a narrow age range, which represents a certain limitation.

The results of our study are in very good agreement with the values reported in the extensive database of pediatric reference values created by the Canadian laboratory initiative on pediatric reference intervals (CALIPER) (*30*). According to this database, reference values for cystatin C for children aged 2 to 19 years, obtained by serum analysis are 0.62 to 1.11 mg/L.

Our results also show that cystatin C values after the first year of life are constant and homogeneous within the entire age interval from 1 to 18 years, which is in accordance with the study by Cai *et al.* (*31*). Some studies further divide samples of children from 1 to 18 years into age groups. For example, a large study by Ziegelasch *et al.*, conducted on a 2926 participants, infants, children, and adolescents, using immunoturbidimetric method, found that the reference cystatin C interval depends on age, as well as on sex and height (*32*). However, variances due to a height are small and limited to certain age/sex categories, so the authors recommend only age and sex specific reference values for cystatin C for kidney function estimation.

On the other hand, a study conducted on an ethnically heterogeneous group of children showed the same dynamics of the dependence of serum cystatin C concentration on age as our research (*31*).

Furthermore, obtained cystatin C values were found to be independent of sex. Data in the CALIPER database indicate a certain difference by sex, although only in the age interval between 1 and 2 years (0.77-1.85 mg/L for male and 0.60-1.20 mg/L for female children) (*30*). Same is with already mentioned study of Ziegelasch *et al.* (*32*). However, numerous studies have shown that cystatin C concentration in children aged 1 to 18 years is independent of sex, which is consistent with the results reported here (*6*, *9*, *28*, *29*, *31*).

We also demonstrated that the determination of cystatin C in serum and the use of the Schwartz equation to predict GFR can provide a simple and reliable way of estimating renal function in children and can contribute to the ability of clinicians to assess kidney function.

In conclusion, the cystatin C reference intervals for Croatian pediatric population according to age were determined. We have confirmed that the cystatin C concentrations in children reach adulthood values at the age of one year. The Schwartz’s formula can be applied to estimate glomerular filtration from cystatin C.

## Data Availability

The data generated and analyzed in the presented study are available from the corresponding author on request.
